# Supplementary material for: MetaRibo-Seq measures translation in microbiomes
Source: Nat Commun. 2020 Jun 29;11:3268. doi: 10.1038/s41467-020-17081-z (PMC7324362; doi:10.1038/s41467-020-17081-z)
Supplement: Supplementary file 10 — Supplementary Data 7 [file 41467_2020_17081_MOESM10_ESM.zip › File2/Confidence_VeryHigh_Taxonomy/391501_out.krona.html]

Javascript must be enabled to view this page.

members
magnitude
magnitudeUnassigned
count
unassigned
taxon
rank

391501\_out

4

2
superkingdom
4

1239
4
phylum

186801
4
class

1
order
4

SRS142712\_contig\_number\_contig-100\_1512.36807
186802

216572
1
family

459786
1
genus

1945593
species

SRS053356\_contig\_number\_1618
1

1

SRS053356\_contig\_number\_contig-100\_20.267535
species
2109688

1

SRS893172\_contig\_number\_14121
species
172733
